# Leveraging Artificial Intelligence and Data Science for Integration of Social Determinants of Health in Emergency Medicine: Scoping Review

**DOI:** 10.2196/57124

**Published:** 2024-10-30

**Authors:** Ethan E Abbott, Donald Apakama, Lynne D Richardson, Lili Chan, Girish N Nadkarni

**Affiliations:** 1 Department of Emergency Medicine, Icahn School of Medicine at Mount Sinai, 1 Gustave L Levy Place, New York, NY, 10029, United States, 1 2122416500; 2 Department of Population Health Science and Policy, Icahn School of Medicine at Mount Sinai, New York, NY, United States; 3 Institute for Health Equity Research (IHER), Icahn School of Medicine at Mount Sinai, New York, NY, United States; 4 Division of Data-Driven and Digital Medicine, Icahn School of Medicine at Mount Sinai, New York, NY, United States; 5 Department of Medicine, Division of Nephrology, Icahn School of Medicine at Mount Sinai, New York, NY, United States; 6 The Charles Bronfman Institute for Personalized Medicine, Icahn School of Medicine at Mount Sinai, New York, NY, United States; 7 Department of Genetics and Genomic Sciences, Icahn School of Medicine at Mount Sinai, New York, NY, United States

**Keywords:** data science, social determinants of health, natural language processing, artificial intelligence, NLP, machine learning, review methods, review methodology, scoping review, emergency medicine, PRISMA

## Abstract

**Background:**

Social determinants of health (SDOH) are critical drivers of health disparities and patient outcomes. However, accessing and collecting patient-level SDOH data can be operationally challenging in the emergency department (ED) clinical setting, requiring innovative approaches.

**Objective:**

This scoping review examines the potential of AI and data science for modeling, extraction, and incorporation of SDOH data specifically within EDs, further identifying areas for advancement and investigation.

**Methods:**

We conducted a standardized search for studies published between 2015 and 2022, across Medline (Ovid), Embase (Ovid), CINAHL, Web of Science, and ERIC databases. We focused on identifying studies using AI or data science related to SDOH within emergency care contexts or conditions. Two specialized reviewers in emergency medicine (EM) and clinical informatics independently assessed each article, resolving discrepancies through iterative reviews and discussion. We then extracted data covering study details, methodologies, patient demographics, care settings, and principal outcomes.

**Results:**

Of the 1047 studies screened, 26 met the inclusion criteria. Notably, 9 out of 26 (35%) studies were solely concentrated on ED patients. Conditions studied spanned broad EM complaints and included sepsis, acute myocardial infarction, and asthma. The majority of studies (n=16) explored multiple SDOH domains, with homelessness/housing insecurity and neighborhood/built environment predominating. Machine learning (ML) techniques were used in 23 of 26 studies, with natural language processing (NLP) being the most commonly used approach (n=11). Rule-based NLP (n=5), deep learning (n=2), and pattern matching (n=4) were the most commonly used NLP techniques. NLP models in the reviewed studies displayed significant predictive performance with outcomes, with F1-scores ranging between 0.40 and 0.75 and specificities nearing 95.9%.

**Conclusions:**

Although in its infancy, the convergence of AI and data science techniques, especially ML and NLP, with SDOH in EM offers transformative possibilities for better usage and integration of social data into clinical care and research. With a significant focus on the ED and notable NLP model performance, there is an imperative to standardize SDOH data collection, refine algorithms for diverse patient groups, and champion interdisciplinary synergies. These efforts aim to harness SDOH data optimally, enhancing patient care and mitigating health disparities. Our research underscores the vital need for continued investigation in this domain.

## Introduction

Medical care, while crucial, contributes to only about 20% of the modifiable factors influencing a population’s health outcomes, while 80% are influenced by genetics, individual behaviors, and socioeconomic factors. The latter two form the social determinants of health (SDOH) [1] that operate at various levels. From macroeconomic policies of nations to public education and housing policies, these structural factors shape resource distribution and societal positions. Consequently, they influence living conditions, access to essential resources, and daily life circumstances, ultimately molding health and health disparities [2]. Every patient’s health trajectory is influenced by SDOH, which can manifest positively (eg, high income, food security) or adversely [3]. The negative aspects can be categorized into social risks, conditions linked to poor health, and social needs, which are individual preferences for assistance [4]. These determinants, especially when adverse, can hinder optimal care and impact clinical outcomes [5]. While not the focus of this review, health-related social needs reflect individual level social needs, and are inextricably related to the impacts and conditions of SDOH.

Emergency medicine (EM) is a unique medical specialty: it can both identify and address adverse SDOH, making it a pivotal setting for intervention. The high prevalence of social needs among emergency department (ED) patients, especially those with low socioeconomic status, housing insecurity, or limited access to care, underscores the potential of ED-based SDOH interventions [6,7]. However, there are significant challenges; comprehensive social risk screenings in the ED are often impractical due to patient volume, acuity, and health system financial constraints. Relying solely on electronic health records (EHRs) is time-consuming and inconsistent. Furthermore, the scattered and unstructured nature of SDOH data in EHRs makes it difficult for ED physicians to identify patients with adverse SDOH [8-10]. However, advancements in techniques in the data sciences provide novel methods to address these issues.

Social informatics, a subfield of medical informatics, refers to the usage of information technology to better harmonize social and health data through improved capture and usage. It bridges the gap between the technical and social worlds, offering insights into how technology and societal factors interplay. The vast possibilities of social informatics lie in its potential to reshape how we understand, interpret, and act on social data in health care settings. By integrating social data with health data, it aims to enhance clinical care and overall health outcomes [11]. Techniques like natural language processing (NLP), artificial intelligence (AI), and machine learning (ML) are being harnessed to extract, use, and model SDOH data effectively [12-14]. AI represents an umbrella term that broadly encompasses computer systems that can achieve human-level performance on cognitive tasks [15], while NLP, a subfield of AI, is a field of computational linguistics that involves analyzing and understanding human language. This entails using a combination of statistical approaches, including ML, to extract structure and meaning from language [16]. Pertinent to this review, ML can also be used as an analytic technique for predictive modeling and better understanding patterns in data sets.

While existing literature has touched upon SDOH in the ED, a comprehensive review focusing on the application of data sciences in this context is lacking. This scoping review aims to map the current literature, pinpoint areas for future research, and highlight the transformative potential of integrating data sciences into EM SDOH research.

## Methods

### Data Sources and Literature Search Strategy

To capture the evolving role of AI and advancing data science techniques for patients seen in the ED, particularly pertaining to SDOH, we searched the literature from 2015 to 2022, a period marked by rapid advancements in AI applications in health care. We included articles from databases such as Medline (Ovid), Embase (Ovid), CINAHL, Web of Science, and ERIC, prioritizing research that melded data science with emergency care settings.

Our search encompassed terms related to SDOH, data science techniques such as ML algorithms, NLP, AI, and EM (see Multimedia Appendix 1 for search terms used). We chose to include EM patient populations to best understand this clinical context. While there is no uniform definition for data science, for this review we have used the definition proposed by Mike and Hazzan [17] that considers data science as a research method by “integration of research tools and methods taken from statistics and computer science that can be used to conduct research in various application domains, such as social science and digital humanities”.

### Article Selection

We focused our review on studies leveraging data science techniques to extract or model SDOH data in EM. Recognizing the paucity of EM-specific research using AI/ML algorithms, we also considered studies on emergency-related conditions that might be seen in other clinical non-ED settings. This included conditions such as opioid use disorder (OUD), HIV, and epilepsy. We intentionally excluded COVID-19 studies, given their unique characteristics and sheer volume of literature. This exclusion ensured a more focused review with current relevance. Two independent reviewers (DA and EA) assessed titles and abstracts for final inclusion. Any disagreements were resolved through joint discussions. We used Covidence (Covidence systematic review software; Veritas Health Innovation), a standardized systematic review software.

### Data Extraction

We extracted data from the selected studies using a standardized form, ensuring uniformity. We captured study objectives, methods, clinical care setting, ML algorithms, modeling approaches, and specified outcomes (see Multimedia Appendix 2). We focused on broad data science techniques, including subfields of AI such as ML algorithms, NLP key SDOH domains, and overall clinical outcomes. While our focus remains descriptive, we abstained from a quality assessment, aligning with standard scoping review guidelines.

## Results

### Overall Study Characteristics

**Figure 1.**
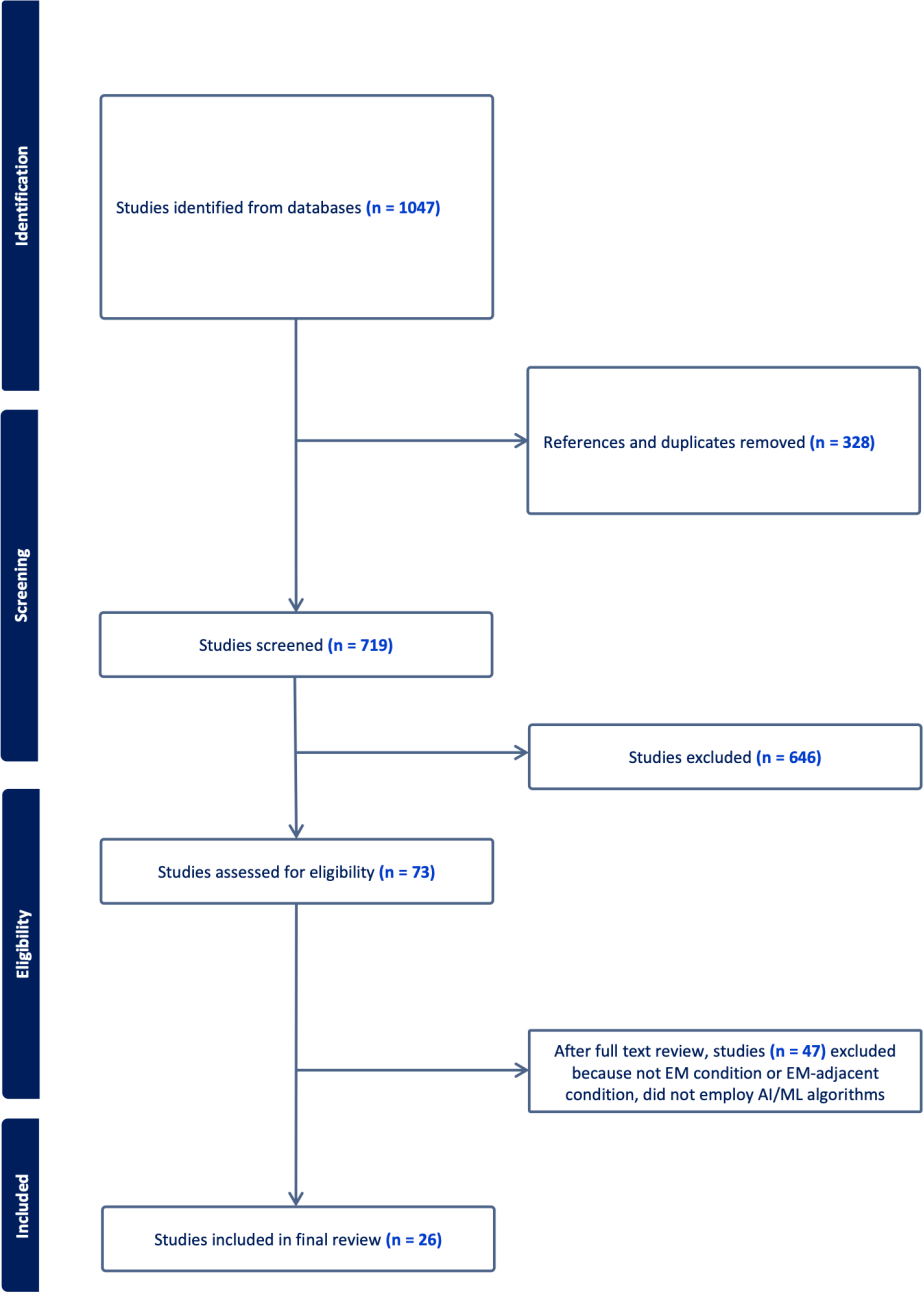
Preferred reporting for systematic reviews and meta-analysis flow chart (PRISMA) diagram: 2015‐2022 search of Medline (Ovid), Embase (Ovid), Cumulative Index to Nursing and Allied Health Literature (CINAHL), Web of Science, and Education Resource Information Center (ERIC).

**Figure 2.**
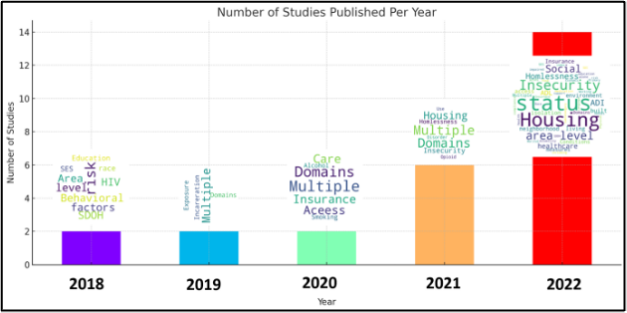
Number of publications by year (2015‐2022) identified in this review. Word clouds show main themes of papers for the corresponding year.

After screening 1047 studies, 26 met our final inclusion criteria (Figure 1 and Multimedia Appendix 1). We excluded a significant number of studies because they did not focus on EM conditions or complaints and did not use AI/ML techniques in the overall approach to the study question. Most studies were published after 2020 (Figure 2) and included patient populations focusing exclusively on the ED (n=9), pediatric patients (n=2), patients treated by emergency medical services (n=2), and US veterans (n=2), among other examples.

### SDOH Domains

Over 60% of studies we identified (n=16) used a broad array of data science techniques such as ML or NLP to use, model, or extract features across more than one of the major SDOH domains, resulting in significant overlap across publications included in this review (Figure 2). These domains included housing insecurity and homelessness, neighborhood and built environment, income and socioeconomic status, employment, family and social support, food insecurity, insurance status and stability, and history of incarceration. While individual level SDOH data was the prevalent unit of analysis, 5 studies used area level data or aggregated measures such as the Social Deprivation Index, Area Deprivation Index, or the Gini coefficient. Housing insecurity and homelessness emerged as the most predominant SDOH domains assessed among 23% of the studies identified (n=6). The domain of neighborhood and built environment was also present across multiple studies and the focus of several publications (n=4). Exposure to or history of incarceration (n=2) as well as OUD (n=2) were also notable.

### Exploration of Emergency Medicine Conditions

The scope of EM clinical conditions and complaints that were studied were broad, including sepsis, acute myocardial infarction, heart failure, asthma, diabetes, chest pain, and epilepsy (Multimedia Appendix 3). Sepsis was the only specific EM condition we identified in more than one study (n=2). Several studies focused on all-cause ED revisits (n=2), “preventable visits” and admissions (n=2), and ED utilization (n=2).

### AI and ML Algorithms

**Figure 3.**
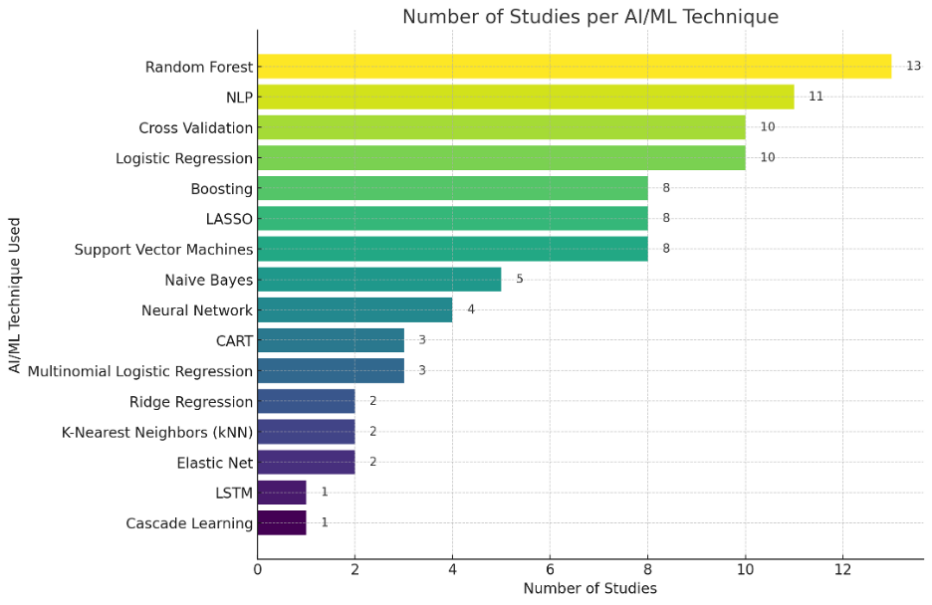
Overall counts of AI/ML algorithms used. AI: artificial intelligence; ML: machine learning; NLP: natural language processing; CART: classification and regression trees; LSTM: long short-term memory.

AI techniques were used in 23 studies, encompassing methods like random forest, classification and regression trees, support vector machines, neural networks, and NLP (Figure 3 and Table 1). Of these, random forest emerged as the most common (n=13), closely followed by NLP (n=11). Key algorithms are discussed in further detail.

**Table 1.** Emergency medicine applications of random forest techniques used for extraction of social determinants of health data.

Category	Instances (n, %)
Identifying and highlighting pivotal SDOH^a^ variables	2 (15.4)
Predictive modeling for potential health trajectories	1 (7.7)
Imputation using random forest techniques	3 (23.1)
Data integrity and robustness evaluation	5 (38.5)
Tree representations	2 (15.4)

a SDOH: Social Determinants Of Health

### Random Forest in SDOH Variable Classification and Data Management

Random forest, an advanced ensemble ML method, was notably present across multiple studies identified in this review. This technique was used to discern and highlight pivotal SDOH variables. Its ability to create predictive models, offering foresight into potential health trajectories based on the subtleties of SDOH indicators was also evident. For example, in “Improving Fairness in the Prediction of Heart Failure Length of Stay and Mortality by Integrating Social Determinants of Health,” the authors used random forest classifiers on sex, ethnoracial, and insurance for study subpopulations, finding differences in underdiagnosis and overdiagnosis of heart failure [18]. The most common use of random forest, however, was to address missing EHR data through imputation, ensuring the integrity and robustness of analyses. Beyond its analytical capabilities, it was also used to create insightful visual representations, offering a comprehensive view into the intricate web of variables, their interactions, and their overarching impact on disease states like acute coronary syndrome, epilepsy, asthma, and OUD.

### NLP for SDOH Data Extraction

**Figure 4.**
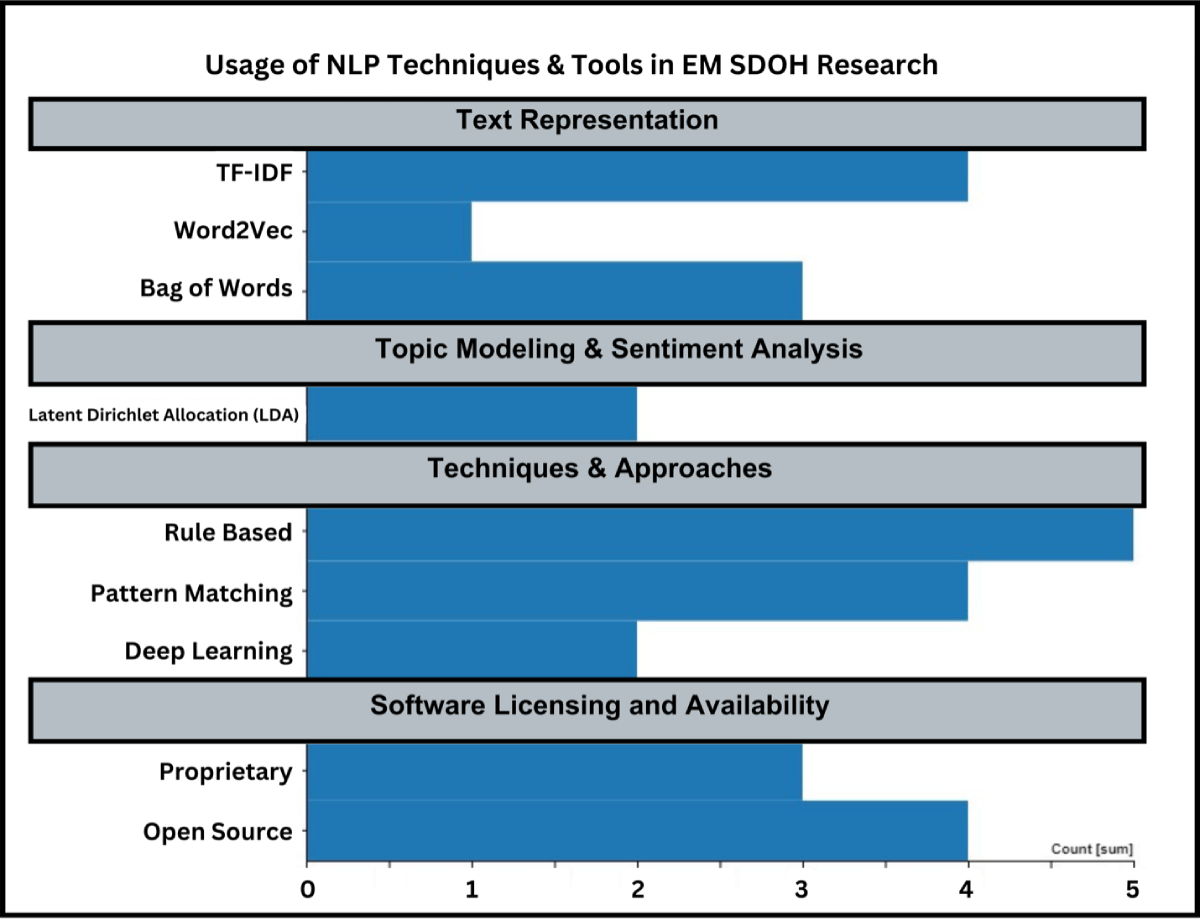
Overall counts of NLP techniques used for extraction of SDOH data including natural language processing (NLP), social determinants of health (SDOH), term frequency-inverse document frequency (TF-IDF) .

The integration of NLP in the realm of EM offers a promising avenue for the precise extraction of SDOH from EHRs. Traditional methodologies, such as manual reviews and rudimentary keyword searches, are increasingly recognized for their inherent limitations, particularly in the context of vast and intricate EHR data sets. Our scoping review elucidated the prevalence of several NLP techniques in the field. Text representation methods like term frequency-inverse document frequency (n=4), Bag of Words (n=3), and Word2Vec (n=1) were prominently used, underscoring their fundamental role in converting textual data into computationally amenable formats (Figure 4). In the realm of topic modeling and semantic analysis, latent Dirichlet allocation was noted in 2 studies, highlighting its potential in discerning latent topics within medical records. Approaches favored rule-based methodologies, found in 5 studies, followed by deep learning (n=2) particularly structures like bidirectional long short-term memory and pattern matching (n=4). From a software perspective, both proprietary (n=5) and open source (n=6) tools were harnessed, reflecting the diverse ecosystem of NLP tools available for research. These NLP methodologies, especially the dominant ones like Bag of Words, term frequency-inverse document frequency, and deep learning structures, have demonstrated notable efficacy. However, to achieve the pinnacle of precision in SDOH data extraction, it is imperative to continually refine these NLP techniques. Collaborative endeavors involving domain experts, iterative model training, and the assimilation of multifaceted data sources are paramount to enhancing the accuracy and relevance of extracted insights.

### Health Information Exchange (HIE) for SDOH Data Aggregation

**Figure 5.**
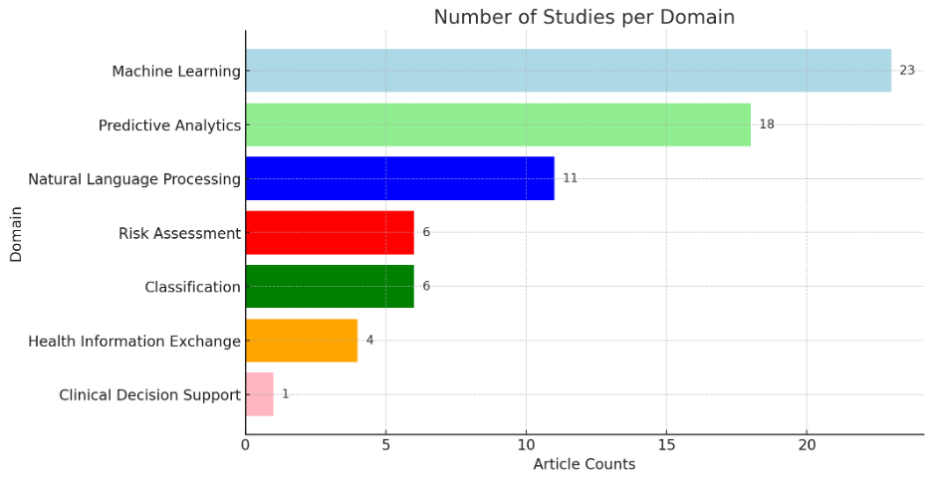
Overall counts for identified articles by domain and/or task.

Health information exchange (HIE), featured in 4 out of 26 studies (Figure 5), aggregates patient data across health care entities, providing a comprehensive view of a patient’s clinical journey. With 40% of patients in one study having encounters at multiple organizations, the importance of HIE in reflecting the distributed nature of ED care becomes evident. HIE can aid in analyzing care transitions and augmenting the sample size and diversity for SDOH research. However, challenges like data sharing, data quality, and privacy regulations need to be addressed. In essence, HIE holds immense potential for EM research, offering both multi-organizational and community-level insights.

### Predictive Applications

ML techniques can be important tools to leverage for clinical outcome predictions, and we identified a significant number of studies in our review. A total of 19 studies, representing a diverse array of ML strategies were identified, each tailored for distinct predictive outcomes. The efficacy of predictive models was a pivotal outcome in 10 studies. Performance metrics for these models, when used as a primary outcome, showcased a range of F1-scores from 0.4 to 0.75, indicating varied precision and recall across studies. These ranged from supervised models like random forest classifiers for acute coronary syndrome predictions to neural networks targeting sepsis-related readmission risks. Notably, ensemble methods were adeptly used to discern primary risk factors for OUDs. Within this realm, NLP proved instrumental, particularly in classification tasks, risk stratification, and fortifying clinical decision-making processes. Beyond this, key outcomes highlighted patterns in ED revisits (n=6), in-hospital mortality (n=2), algorithmic bias (n=1), and mismatches between physician annotations and claims data (n=1).

### Data Quality, Privacy, and Algorithmic Bias

In the comprehensive spectrum of studies analyzed, an intriguing observation surfaced: a noticeable gap in the examination of data quality and privacy concerns within the realm of SDOH and EM. Despite sifting through a multitude of research articles, we did not encounter any study that directly tackled these pivotal issues. This omission underlines the potential vulnerabilities in the application of AI and ML techniques, especially when handling sensitive patient data. Furthermore, a singular study broached the topic of algorithmic bias, a topic of paramount importance given the potential repercussions on health care outcomes and equity. The underrepresentation of these themes hints at uncharted territories that warrant meticulous exploration in future research endeavors, ensuring that the integration of AI and ML in EM is both robust and ethically sound.

## Discussion

### Principal Findings

The leveraging of SDOH data within EM research and clinical care is pivotal for gaining new insights, improving patient outcomes, and optimizing health care delivery. Our scoping review highlights the transformative role of data science, chiefly AI/ML, and the subdiscipline of NLP, in improving SDOH data integration and modeling within EM. Emerging from our review is an increase toward using data science to harness, operationalize, and model SDOH in emergency care settings. This progression signifies a shift: a pivot toward a comprehensive, data-infused approach that addresses not just emergent conditions but also the intricate web of social and economic determinants impacting health. NLP excels at extracting SDOH information from the unstructured text of EHRs, while ML’s predictive strength can transform these insights into actionable predictions. Such models, equipped with SDOH data, can catalyze precision interventions, potentially identifying mechanisms for ED revisits, in-hospital mortality, and readmissions.

Delving deeper into the SDOH domains, housing insecurity and the neighborhood environment emerged as primary determinants, witnessing significant attention across the studies. Their frequent appearance in the research landscape underscores their profound impact on health outcomes within emergency settings. While these domains were at the forefront, other determinants like education, employment, and social networks were also featured, albeit to a lesser extent. The emphasis on these SDOH domains, especially housing insecurity, suggests a pressing need for targeted interventions and policies within emergency care settings. As the health care sector continues to evolve, understanding these predominant SDOH domains and harnessing the power of data science will be pivotal in offering a more holistic, patient-centric approach to emergency care.

Our comprehensive review, while offering insights, bears certain limitations warranting acknowledgment. The time frame for our study, confined to 2015-2022, captures most contemporary advancements but might inadvertently omit foundational studies predating this period, potentially offering evolutionary insights. While we highlighted NLP and other ML techniques, the vast expanse of data science boasts other emerging tools such as the recent and rapid development of large language modeling (LLM) such as ChatGPT (OpenAI), which were absent in our review. The potential applications of LLM in harnessing and modeling SDOH within the EM setting are rapidly emerging and will likely expand the possibilities for improving health outcomes and disparities.

Second, the encompassing scope of our review, spanning diverse SDOH domains and emergency conditions, enriches the study’s comprehensiveness. Yet, the scope also poses challenges in distilling specific conclusions regarding the utility of data science techniques across distinct SDOH or EM conditions. Comparative analysis across studies was hampered by the varied outcome measures adopted. Although a significant number focused on the performance of predictive models, this only scratches the surface of data science’s potential impact on SDOH within EM. Lastly, our review refrained from assessing the methodological quality of the incorporated studies. This approach aligns with scoping review guidelines but omits considerations of each study’s methodological soundness during our synthesis.

Amidst these intricacies and challenges, there are still other pressing concerns. Data quality, privacy concerns, and algorithmic biases are potential hurdles that merit attention. In particular, the limited exploration and assessment of algorithmic bias in our reviewed studies, given its potential to perpetuate health care disparities, suggests an urgent avenue for further investigation. Without careful assessment of algorithmic biases, there exists the potential for reinforcing racial and ethnic discrimination and institutional racism. Only one study we identified in our review specifically assessed ML model bias and fairness in the context of heart failure outcomes [18]. ML algorithmic biases are critical to address in the context of SDOH research, as prior studies have demonstrated the potential for reinforcement of pre-existing racial, ethnic, and socioeconomic disparities [19].

### Future Works and Recommendations

#### Potential Areas of Exploration

Our review has illuminated the significance of certain domains like housing insecurity, within the context of SDOH in EM. However, the vast landscape of SDOH offers numerous other domains that remain relatively unexplored:

1. **Education:** Investigating the role of educational attainment and access to quality education can provide insights into its impact on health outcomes. For instance, understanding how literacy levels influence patient adherence to medical advice in emergency settings could be pivotal. AI techniques could be used to better understand the complexity of both current educational attainment and future or desired educational needs through careful extraction or modeling of data.
2. **Employment:** Employment status, job security, economic mobility, and workplace conditions can have profound effects on mental and physical health. Exploring these factors can shed light on stress-related conditions or injuries that present in EDs.
3. **Social networks:** The influence of social support systems, community engagement, and familial ties can play a crucial role in patient recovery and mental well-being. Delving into these aspects can offer a holistic view of a patient’s environment and its implications for health.

With these potential areas in mind, it becomes evident that a multi-faceted approach to SDOH within EM is the way forward. Building on these areas of exploration, we propose several recommendations to harness the full potential of SDOH in EM.

#### Recommendations

1. **Establishing gold standard metrics:** For the evolution and standardization of emergency SDOH research, it is essential to define and adopt gold standard metrics. These metrics should be robust, universally accepted, and tailored to capture the nuances of SDOH in emergency settings. Collaborative efforts among researchers, clinicians, and policy makers should be made to create these benchmarks.
2. **Innovative data capture and implementation:** The high-paced nature of emergency settings necessitates innovative solutions for capturing SDOH data. Leveraging AI-assisted tools or predictive algorithms based on existing patient data could offer one approach. Creating automated workflows to allow for capture and implementation of SDOH data at the patient encounter level will be important for addressing social risks and needs.
3. **Algorithmic innovation:** The prominence of ML and NLP in our findings suggests a horizon brimming with algorithmic advancements and adaptation for EM. As these tools evolve and new tools emerge, crafting and evaluating interventions tailored to specific SDOH is crucial.
4. **Connecting SDOH with clinical outcomes:** Beyond identifying SDOH, understanding their tangible impact on patient outcomes is vital. A concerted effort in this direction can revolutionize our care approach. The intersection of SDOH and clinical outcomes is recognized by the Centers for Medicare and Medicaid Services, which requires health care systems to screen for key SDOH domains.
5. **Interdisciplinary collaboration:** The confluence of expertise, from clinicians to data scientists, will be instrumental in harnessing the full potential of SDOH data.
6. **Addressing algorithmic bias:** As we increasingly rely on algorithms, it is imperative to ensure they are free from biases that could perpetuate or exacerbate health disparities. Rigorous testing, validation, and refinement of algorithms, with a focus on fairness and equity, should be prioritized.

### Conclusions

This scoping review underscores the transformative potential of data science in elevating the understanding and application of SDOH within EM. Through the adept integration of data science methodologies, particularly ML and NLP, we are poised to redefine the way SDOH data is adopted within EM. This offers a broader and more data-informed approach to influencing critical patient outcomes. The literature landscape indicates a promising embrace of this cross-disciplinary synergy, manifesting in an increasing number of studies that deploy data science methodologies to unearth, interpret, or model SDOH within emergency care contexts. Such a trajectory not only affirms the growing acknowledgment of these methodologies’ efficacy but also underlines the health care sector’s commitment to delivering more holistic care.

Nevertheless, our review also pinpoints avenues that warrant deeper exploration. Despite the expansive focus on various SDOH domains, certain determinants like housing insecurity and the neighborhood environment have garnered disproportionate attention. A more balanced exploration across SDOH domains would provide a richer, more comprehensive insight into their collective and individual impacts on patient trajectories. Moreover, while the current trend leans heavily on ML and NLP, there exists a vast expanse of data science techniques yet to be fully leveraged like LLM. Diving into these untapped methodologies might further refine our capabilities in SDOH identification and intervention.

In conclusion, the fusion of data science with EM marks the dawn of a new health care epoch. It envisions a future where EDs transcend their traditional roles, evolving into hubs that address the foundational SDOH challenges within communities. As we navigate this promising trajectory, the potential to revolutionize EM and fortify patient-centric care is immense.

## Supplementary material

10.2196/57124 Multimedia Appendix 1 Finalized search strategies.

10.2196/57124 Multimedia Appendix 2 Finalized complete list of literature included in the review.

10.2196/57124 Multimedia Appendix 3 Emergency medicine conditions word cloud. Size of word cloud represents aggregated counts of terms from titles of articles identified for this review (Python (version 3.7), matplot library).

10.2196/57124 Checklist 1 PRISMA (Preferred Reporting Items for Systematic Reviews and Meta-Analysis flow chart) checklist.

## Abbreviations

AI: artificial intelligence
ED: emergency department
EHR: electronic health record
EM: emergency medicine
HIE: health information exchange
LLM: large language modeling
ML: machine learning
NLP: natural language processing
OUD: opioid use disorder
SDOH: social determinants of health
