# Supplementary material for: Leveraging Artificial Intelligence and Data Science for Integration of Social Determinants of Health in Emergency Medicine: Scoping Review
Source: JMIR Med Inform. 2024 Oct 30;12:e57124. doi: 10.2196/57124 (PMC11539921; doi:10.2196/57124)
Supplement: Multimedia Appendix 1 [file medinform-v12-e57124-s001.docx]

**Supplemental Table 1**: Finalized search strategies

Embase Classic+Embase

| 1 | social determinants of health/ |
| --- | --- |
| 2 | ("Social determinants of health" or "Social risk" or "social need*").mp. |
| 3 | 1 or 2 |
| 4 | natural language processing/ |
| 5 | (Natural language processing or NLP).mp. [mp=title, abstract, heading word, drug trade name, original title, device manufacturer, drug manufacturer, device trade name, keyword heading word, floating subheading word, candidate term word] |
| 6 | machine learning/ |
| 7 | machine learning.mp. |
| 8 | artificial intelligence/ |
| 9 | ("artificial intelligence" or AI).mp. |
| 10 | clinical decision support system/ |
| 11 | Clinical decision support system*.mp. |
| 12 | Precision Medicine.mp. |
| 13 | predictive analytics.mp. |
| 14 | predictive model*.mp. |
| 15 | m-health.mp. |
| 16 | or/4-15 |
| 17 | emergency medicine/ |
| 18 | hospital emergency service/ |
| 19 | ("emergency medicine" or "emergency department*" or "emergency room*" or "emergency unit*" or "emergency ward*" or "emergency service*").mp. |
| 20 | 17 or 18 or 19 |
| 21 | 3 and 16 and 20 |

Cochrane CENTRAL Search

| ID | Search |
| --- | --- |
| #1 | MeSH descriptor: [Social Determinants of Health] this term only |
| #2 | ("Social determinants of health" OR "Social risk" OR "social need*"):ti,ab,kw |
| #3 | MeSH descriptor: [Natural Language Processing] this term only |
| #4 | ("Natural language processing" OR NLP):ti,ab,kw |
| #5 | MeSH descriptor: [Machine Learning] this term only |
| #6 | (machine learning):ti,ab,kw |
| #7 | MeSH descriptor: [Artificial Intelligence] this term only |
| #8 | ("artificial intelligence" OR AI):ti,ab,kw |
| #9 | MeSH descriptor: [Decision Support Systems, Clinical] this term only |
| #10 | ("Clinical decision support system*"):ti,ab,kw |
| #11 | MeSH descriptor: [Precision Medicine] this term only |
| #12 | (precision medicine):ti,ab,kw |
| #13 | ("predictive analytics"):ti,ab,kw |
| #14 | ("predictive model*"):ti,ab,kw |
| #15 | (m-health):ti,ab,kw |
| #16 | MeSH descriptor: [Emergency Medicine] this term only |
| #17 | MeSH descriptor: [Emergency Service, Hospital] this term only |
| #18 | (emergency medicine OR emergency department* OR emergency room* OR emergency unit* OR emergency ward* OR emergency service*):ti,ab,kw |
| #19 | {OR #1-#2} |
| #20 | {OR #3-#15} |
| #21 | {OR #16-#18} |
| #22 | #19 AND #20 AND #21 |
