# Supplementary material for: Leveraging Artificial Intelligence and Data Science for Integration of Social Determinants of Health in Emergency Medicine: Scoping Review
Source: JMIR Med Inform. 2024 Oct 30;12:e57124. doi: 10.2196/57124 (PMC11539921; doi:10.2196/57124)
Supplement: Multimedia Appendix 2 [file medinform-v12-e57124-s002.docx]

**Supplemental Table 2: Finalized Complete List of Literature Included in the Review**

| **Article Title** | **Authors** | **Journal** | **Publication Year** |
| --- | --- | --- | --- |
| Inclusion of social determinants of health improves sepsis readmission prediction models | Amrollahi et al. | JAMIA | 2022 |
| Information Extraction From Electronic Health Records to Predict Readmission Following Acute Myocardial Infarction: Does Natural Language Processing Using Clinical Notes Improve Prediction of Readmission? | Brown et al. | JAHA | 2022 |
| ReHouSED: A novel measurement of Veteran housing stability using natural language processing | Chapman et al. | Journal of Biomedical Informatics | 2021 |
| Neighborhood-level Social Determinants of Health Improve Prediction of Preventable Hospitalization and Emergency Department Visits beyond Claims History | Chi et al. | Population Health Management | 2021 |
| Development of a homelessness risk screening tool for emergency department patients | Doran et al. | HSR | 2022 |
| A Statistical-Learning Model for Unplanned 7-Day Readmission in Pediatrics | Ehwerhemuepha | Hospital Pediatrics | 2020 |
| Your neighborhood matters: A machine-learning approach to the geospatial and social determinants of health in 9-1-1 activated chest pain | Faramand et al. | Research in Nursing and Health | 2022 |
| Using Clinical Notes and Natural Language Processing for Automated HIV Risk Assessment | Feller et al. | JAIDS | 2018 |
| Use of Stratified Cascade Learning to predict hospitalization risk with only socioeconomic factors | Filikov et al. | Journal of Biomedical Informatics | 2020 |
| Predicting Opioid Use Disorder and Associated Risk Factors in a Medicaid Managed Care Population | Gao et al. | American Journal of Managed Care | 2021 |
| Predicting frequent emergency department use among children with epilepsy: A retrospective cohort study using electronic health data from 2 centers | Grinspan et al. | Epilepsia | 2017 |
| Multiple Electronic Health Record-Based Measures of Social Determinants of Health to Predict Return to the Emergency Department Following Discharge | Hall et al. | Population Health Management | 2022 |
| Classifying social determinants of health from unstructured electronic health records using deep learning-based natural language processing | Han et al. | Journal of Biomedical Informatics | 2022 |
| Community-based participatory research application of an artificial intelligence-enhanced electrocardiogram for cardiovascular disease screening: A FAITH! Trial ancillary study | Harmon et al. | American Journal of Preventative Cardiology | 2022 |
| Development and assessment of a natural language processing model to identify residential instability in electronic health records' unstructured data: A comparison of 3 integrated healthcare delivery systems | Hatef et al. | JAMIA | 2022 |
| Measuring the Value of a Practical Text Mining Approach to Identify Patients With Housing Issues in the Free-Text Notes in Electronic Health Record: Findings of a Retrospective Cohort Study | Hatef et al. | Frontiers in Public Health | 2021 |
| Improving Fairness in the Prediction of Heart Failure Length of Stay and Mortality by Integrating Social Determinants of Health | Li et al. | Circulation Heart Failure | 2022 |
| Developing a Model to Predict High Health Care Utilization Among Patients in a New York City Safety Net System | Li et al. | Medical Care | 2023 |
| Using Natural Language Processing to Examine Social Determinants of Health in Prehospital Pediatric Encounters and Associations with EMS Transport Decisions | Lowery et al. | Prehospital Emergency Care | 2023 |
| Methods for development and application of data standards in an ontology-driven information model for measuring, managing, and computing social determinants of health for individuals, households, and communities evaluated through an example of asthma | Rousseau et al. | Journal of Biomedical Informatics | 2022 |
| Identification of social determinants of health using multi-label classification of electronic health record clinical notes | Stemerman et al. | JAMIA Open | 2021 |
| Screening for Social Determinants of Health: Active and Passive Information Retrieval Methods | de Ramirez et al. | Population Health Management | 2022 |
| Natural language processing and machine learning of electronic health records for prediction of first-time suicide attempts | Tsui et al. | JAMIA Open | 2021 |
| Prediction of emergency department revisits using area-level social determinants of health measures and health information exchange information | Vest et al. | International Journal of Medical Informatics | 2019 |
| Measuring Exposure to Incarceration Using the Electronic Health Record | Wang et al. | Medical Care | 2019 |
| Comparison between machine learning methods for mortality prediction for sepsis patients with different social determinants | Wang et al. | BMC Medical Informatics and Decision Making | 2022 |
