# Supplementary figures and images for: Leveraging Artificial Intelligence and Data Science for Integration of Social Determinants of Health in Emergency Medicine: Scoping Review
Source: JMIR Med Inform. 2024 Oct 30;12:e57124. doi: 10.2196/57124 (PMC11539921; doi:10.2196/57124)

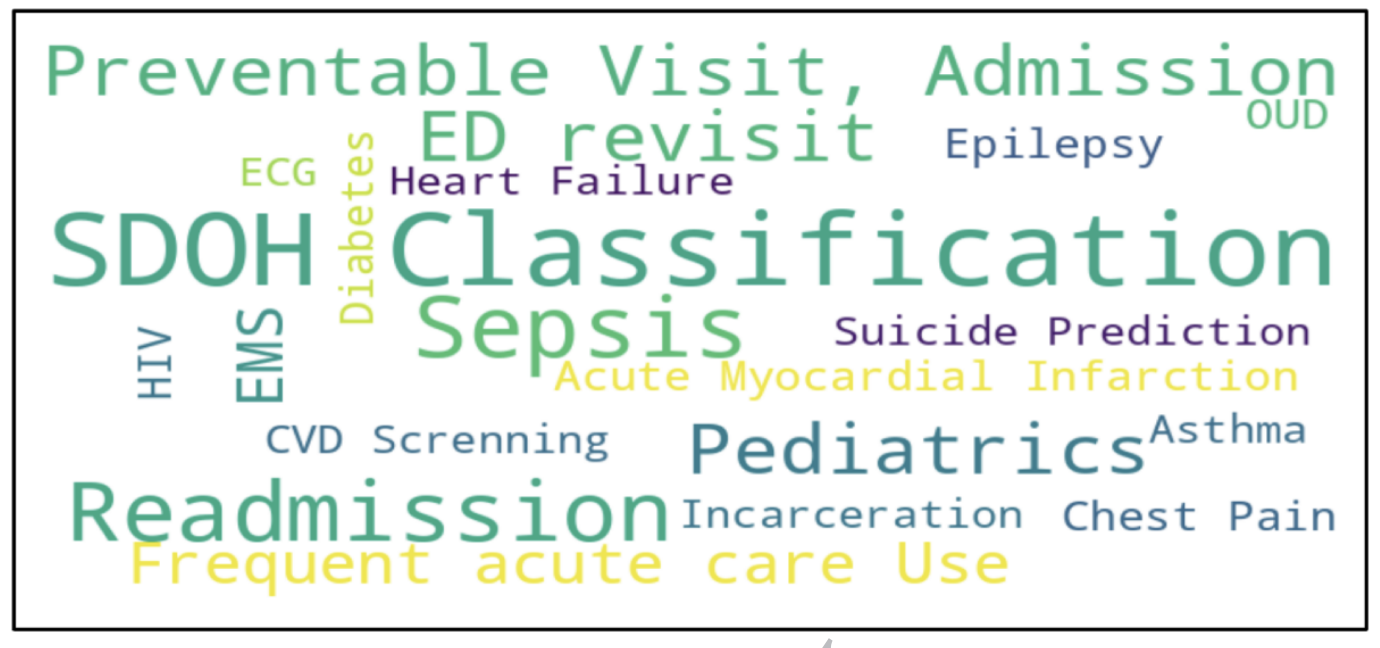

Supplement: Multimedia Appendix 3 [file medinform-v12-e57124-s003.png]
